# Antibacterial Activity of Sodium Hypochlorite Gel versus Different Types of Root Canal Medicaments Using Agar Diffusion Test: An In Vitro Comparative Study

**DOI:** 10.1155/2020/6483026

**Published:** 2020-11-28

**Authors:** Mohamed El Sayed, Nikta Ghanerad, Fatemeh Rahimi, Mahin Shabanpoor, Zeinab Shabanpour

**Affiliations:** ^1^Ajman University, College of Dentistry, Restorative Dentistry Department, P.O. Box 346, Ajman, UAE; ^2^Mansoura University, Faculty of Dentistry, Endodontic Department, El-Gomhoureya Street, El-Mansoura city, 35516 El-Dakahleya, Egypt

## Abstract

**Aim:**

This study aimed to evaluate the antibacterial effect of sodium hypochlorite gel and four types of intracanal medicaments.

**Materials and Methods:**

The agar diffusion method was used to evaluate the antibacterial effect of five medicaments (sodium hypochlorite gel (NaOCl), chlorhexidine gel (CHX), calcium hydroxide paste (CH), Ledermix, and Diapex plus) against *Enterococcus faecalis* (*E. faecalis*), *Staphylococcus aureus* (*S. aureus*), and *Escherichia coli* (*E. coli*). The zone of inhibition around each medicament was measured in millimeters, after 48 hours of incubation at 37°C. The antibacterial effects of medicaments against each microbial strain and the sensitivity of microorganisms towards each medicament were compared using the one-way ANOVA and Games–Howell post hoc tests. The level of significance was set to *p* < 0.05.

**Results:**

All medicaments showed variable inhibition zones for all bacterial strains except Diapex Plus which showed no antibacterial activity. NaOCl gel exhibited the most significant inhibition zones for all bacterial strains followed by CHX gel, Ledermix, and CH. However, the effect of CHX and CH paste against *S. aureus* was statistically similar, while the effect of CH against *E. faecalis* was significantly higher than the Ledermix.

**Conclusion:**

Sodium hypochlorite gel displayed the highest antibacterial activity among tested medicaments and can be recommended as a potent intracanal medicament. Chlorhexidine gel showed a significantly higher antibacterial effect when compared with Ledermix and calcium hydroxide. Calcium hydroxide demonstrated stronger antibacterial activity against *E. faecalis* than Ledermix. Diapex Plus exhibited no antibacterial effect.

## 1. Introduction

Bacteria and their toxic byproducts are the main causes of pulpal and periapical diseases [1]. The most important goals of successful endodontic treatment are to eliminate the microorganisms confined in the root canal system and prevent the regrowth of residual microorganisms [2]. Infected root canals have a complex microbial flora consisting of cocci, rods, spirochetes, filaments, and sometimes fungi that are typically found together in endodontic flare-ups and cases with posttreatment apical periodontitis [3, 4]. Facultative anaerobic bacteria such as *Enterococcus faecalis* (*E. faecalis*) and *Staphylococcus aureus* (*S. aureus*) have been thought to be the most resistant species in the oral cavity that may cause root canal treatment failure [5, 6]. *E. faecalis* can survive in root canals as a single organism without the support of the other bacteria [7]. *S. aureus* can remain viable in dentinal tubules for prolonged periods because of their resistance to drying and temperature changes [8]. *Escherichia coli* (*E. coli*) is an anaerobic Gram-negative bacillus that is commonly associated with persistent periapical infections [9], and its endotoxins play an important role in the induction and perpetuation of periradicular inflammatory lesions of experimental cats [10].

Different instrumentation techniques and irrigation protocols have been recommended for cleaning and disinfecting the root canal system, especially in cases with severe endodontic infections [11]. However, due to the structural complexity of root canals and the limitations of irrigation solutions, it is difficult to fully remove microorganisms from the root canal system [12, 13]. Furthermore, the deep penetration of microorganisms into dentinal tubules makes their elimination difficult [14, 15]. Therefore, the use of an intracanal medicament with broad-spectrum antibacterial activity was necessary to complete disinfection of the prepared root canal system, especially for cases with persistence apical periodontitis [14, 15].

Diverse endodontic medicaments are existing with a variable degree of antimicrobial activity, such as nonsetting calcium hydroxide [Ca (OH)_2_] and chlorhexidine gluconate (CHX). Ledermix is currently a popular root canal medicament consisting of 1% triamcinolone acetonide (corticosteroid) and 3.2% demeclocycline–calcium (tetracycline antibiotic) in a polyethylene glycol base. It has an anti-inflammatory function to alleviate pain associated with symptomatic apical periodontitis and prevent acute exacerbation of chronic apical periodontitis [15, 16]. Diapex Plus (DiaDent, Seoul, Korea) is an alternative intracanal medicament recently launched on the market. It is a premixed paste made up of iodoform (40.4%), Ca (OH)_2_ (30.2%), and hydrophobic silicone oil (22.4%). According to the manufacturer, this product has a strong antibacterial effect and can be used to disinfect the root canals and to treat periapical lesions of endodontic origin. Sodium hypochlorite (NaOCl) gel is available on the market as a Clorox bleaching pen gel (Clorox, USA) for cleaning cloths. The composition of this bleaching material as follows: boehmite (3–7%), sodium hypochlorite (0.5–2%), sodium silicate (0.5–1.5%), and sodium petroleum sulfonate (0.5–1.5%). However, as a trade secret, the manufacturer has withheld the exact percentage concentration of sodium hypochlorite [17]. The NaOCl gel can be used as intracanal medication and lubricant during the root canal instrumentation. As far as our knowledge is concerned, no previous studies have been conducted to determine the antimicrobial activity of sodium hypochlorite gel as an intracanal medicament.

Antimicrobial effectiveness of endodontic materials can be tested either in vitro or in vivo. Agar diffusion test is still the most widely used in vitro tool for evaluating the antimicrobial activity of these materials, despite its drawbacks such as lack of standardization of inoculum density, adequate culture medium, agar viscosity, plate storage conditions, size and number of specimens per plate, and time and temperature of incubation [18]. This test is simple to use and inexpensive and preserves the chemical properties of the materials tested [19]. Numerous studies on the bactericidal effect of currently available intracanal medicaments are contradictory and incomplete, and little studies have been performed on the antibacterial activity of sodium hypochlorite gel. Therefore, this study aimed to use the agar diffusion test to evaluate and compare the antibacterial activity of sodium hypochlorite gel, calcium hydroxide paste, chlorohexidine gel, Ledermix, and Diapex Plus against *S. aureus*, *E. faecalis*, and *E. coli*. The null hypothesis of the current study is that there are no significant differences between the antibacterial effects of experimental medicaments against all selected microorganisms.

## 2. Materials and Methods

### 2.1. Experimental Intracanal Medicaments

Five intracanal medicaments were tested: sodium hypochlorite gel (Clorox Bleach Pen Gel, Clorox, USA), 2% chlorhexidine gel (Conspsis Scrub, Ultradent Products Inc., USA), nonsetting calcium hydroxide paste (Metapaste, Meta Biomed Co., Ltd, Korea), Ledermix (Riemser Pharma GMH, Germany), and DiaPex Plus (DiaDent Group, Korea).  Table 1 shows the compositions of the selected intracanal medicaments and their manufacturers.

### 2.2. Experimental Microorganisms

The antimicrobial activity of the experimental medicaments has been tested against the following microorganisms: *S. aureus*, *E. faecalis*, and *E. coli*.  Table 2 shows the type, source, morphotype, and manufacturer of each selected microorganism.

### 2.3. Inoculum Suspension Preparation

All strains have grown aerobically from frozen stock cultures in brain-heart infusion broth (BHI-Difco Laboratories, Detroit MI) at 37°C for a 24-hour incubation period and checked for microbial growth by turbidity changes. The turbidity of the broth culture for each microbial strain was then diluted using sterile saline until obtaining a suspension with 0.5 turbidities on the McFarland scale.

### 2.4. Agar Diffusion Test

Agar well diffusion tests were performed in 90 mm diameter Petri dishes containing Mueller Hinton Agar (BBL 211438 Becton Dickinson, Sparks, MD, USA) to a depth of 4 mm for all bacterial strains except *E. faecalis*, for which sheep blood agar medium was used (Merck, Darmstadt, Germany). Two agar plates were used to test the antimicrobial activity of the medicaments against one microbial strain. The top surface of each agar plate was flooded with 200 *μ*l inoculum suspension of the selected microbial strain and then dried at 37°C for 15 minutes. In the first agar plate, two equidistance wells of 5 mm diameter and 4 mm depth were punched using a sterile glass Pasteur pipette, while, in the second agar plate, three equidistance wells of similar diameters and depths were punched. A sterile pipette was used to place 50 *μ*l of each medication within each plate's respective well. The sodium hypochlorite gel and calcium hydroxide paste have been put in the wells of the first plate. Diapex Plus, Ledermix, and chlorohexidine gel have been put in the wells of the second plate. The plates were kept at room temperature for two hours to obtain the prediffusion of the medicament through the agar medium and then were incubated for 48 hours under aerobic conditions at 37°C. The entire experiment was replicated ten times for each bacterial strain, for which five all experimental medicaments were tested.

### 2.5. Negative and Positive Growth Controls

#### 2.5.1. Negative Growth Controls (Three Agar Plates)

In the experimental plates, two agar plates were prepared but without microorganisms and the experimental medicaments were placed in the prepared wells. The third plate had neither bacteria nor sealer.

#### 2.5.2. Positive Growth Controls (Eight Agar Plates)

To ensure that the microbial life cycle did not become inactive during the experimental study, each two agar plates were streaked with an individual test microorganism only without medicaments.

### 2.6. Measuring the Size of Inhibition Zones

**Figure 1:**
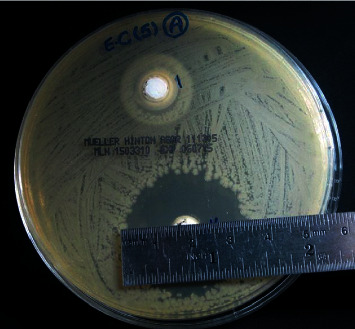
Measuring the inhibition zone using a metal ruler.

Growth inhibition zones around each medicament were evidenced by the lack of bacterial colonization (clearing of agar) adjacent to each agar well. The most uniform diameter segment of the inhibition zone was measured with an endodontic millimeter ruler (Hu-Friedy Mfg. Co., Chicago, IL 60618-5935, USA), and the 6 mm diameter of the well was included in the measurement (Figure 1). All of the measurements above this value were considered indicative of significant inhibition of the bacterial growth. The inhibition zones with wide diameter were interpreted to indicate a higher antibacterial activity of the involved medicaments. At the end of the experiment, ten measurements for each inhibition zone around each medicament and against one type of microbial strain were obtained.

### 2.7. Statistical Analysis

Data were analyzed using version 20 of the SPSS software program (IBM, Armonk, NY, USA). The antibacterial effects of medicaments against each microbial strain and the sensitivity of microorganisms towards each medicament were compared using the One-way ANOVA and Games–Howell post hoc tests. *P* value < 0.05 was considered statistically significant.

## 3. Results

**Figure 2:**
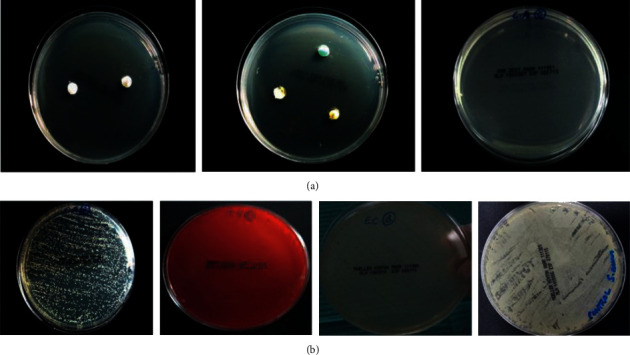
Agar plates for growth controls: (a) Negative growth controls; (b) Positive growth controls.

**Figure 3:**
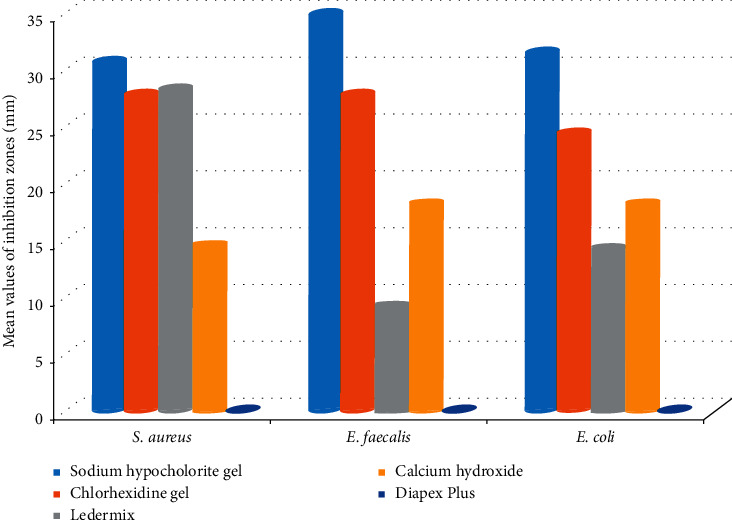
Mean values of the zone diameters of microbial growth inhibition according to the tested medicaments.

**Figure 4:**
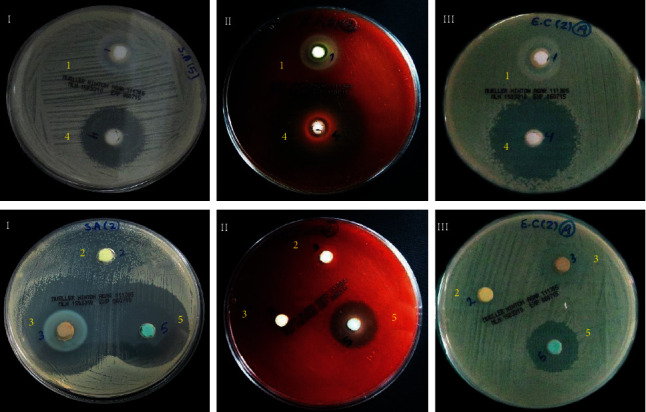
Zones of microbial growth inhibition around each experimental medicament for all tested microbial strains (Medicaments: 1: calcium hydroxide; 2: Diapex Plus; 3: Ledermix; 4: sodium hypochlorite gel; 5: CHX gel; Microbial strains: (I) *S. aureus*; (II) *E. faecalis*; (III) *E. coli*).

There was no bacterial growth in the negative control plates, whereas the positive control plates showed noticeable and even bacterial growth (Figure 2). The mean diameters of inhibition zones developed against the tested microorganisms by the medications are shown in Table 3 and illustrated in Figure 3. The experimental medicaments showed variable diameters of inhibition zones against the tested microorganisms (Figure 4) except for the Diapex Plus that did not display any antibacterial activity against tested microorganisms.

The results of the one-way ANOVA test showed significant differences between the mean values of inhibition zones diameters produced by the tested medications against each bacterial strain (*P* < 0.05). Sodium hypochlorite gel and chlorhexidine gel had the largest inhibition zone diameters against all microorganisms. However, no significant difference was observed between the chlorohexidine gel and Ledermix regarding their antimicrobial effect against *S. aureus*. The antimicrobial activity of calcium hydroxide against *E. faecalis* was significantly higher than Ledermix and its efficacy against *E. coli* and *S. aureus* was significantly less than Ledermix (*P* < 0.05).

It was necessary to evaluate the sensitivity of microorganisms towards each medicament. There were significant differences between the tested microorganisms regarding their sensitivity towards each medicament. The highest inhibition zone around sodium hypochlorite gel occurred with *E. faecalis* (34.87 ± 1.31 mm), followed in descending order by *E. coli* (31.43 ± 2.14 mm) and *S. aureus* (30.68 ± 1.81 mm). The highest inhibition zone with chlorohexidine gel occurred with *S. aureus* (27.83 ± 1.02 mm) followed by *E. coli* (24.40 ± 0.82 mm) and *E. faecalis* (20.93 ± 077 mm).

Regarding the Ledermix, the most extensive inhibition zone occurred with *S. aureus* (28.25 ± 1.87 mm), followed by *E. coli* (14.23 ± 0.53 mm) and *E. faecalis* (9.05 ± 0.75 mm). Nonsetting calcium hydroxide resulted in the most significant zone of inhibition with *E. faecalis* (18.15 ± 0.51 mm), followed in descending order by *S. aureus* (14.45 ± 1.01 mm) and *E. coli* (12.27 ± 1.13 mm).

## 4. Discussion

The interest of researchers for studying antimicrobial properties of root canal irrigating solutions and medicaments has increased over the years, as they become more aware of the significance of the removal of microorganisms from the root canal system [2, 20]. In the current study, the species of microorganisms were chosen to represent microorganisms typically isolated from untreated and previously treated infected root canal systems such as *E. faecalis* [5, 6], *E. coli* [9, 10, 21], and *S. aureus* [5, 6].

Intracanal medicaments have a long history of use as interim appointment dressings to reduce interappointment pain, to decrease the number of microorganisms, and prevent their regrowth and to render the canal contents inert [15]. Evaluation of the antibacterial effectiveness of different intracanal medicaments is needed to understand clearly the role of these medicaments in the treatment of apical periodontitis with variable types of microorganisms.

In this research, calcium hydroxide paste was chosen, as it has antimicrobial properties and mineralization induction capability [22]. However, it has limited action against certain microorganisms, particularly *E. faecalis* [23] and biofilms [24]. In addition, some researchers questioned the effectiveness of calcium hydroxide in reducing microbial numbers, even after prolonged contact with the root canal [13]. To overcome the antimicrobial limitation of calcium hydroxide, some manufacturers have added iodoform to increase its action. Diapex Plus is one of those combinations and was used in the current study as there is little knowledge about its antimicrobial activity. Chlorhexidine gel was another root canal medicament that was selected in this study as it has an excellent antimicrobial activity [25, 26]. It can be used in the treatment of inflammatory root resorption [16] and did not affect the apical seal of the root canal [27, 28]. Some authors suggested the use of 2% chlorhexidine gel as a root canal medication instead of calcium hydroxide [29]. However, it is not an effective intracanal barrier [30] and may reduce the success of root canal treatment [31]. Corticosteroid and antibiotic combinations have been used as an intracanal medicament due to their anti-inflammatory action [32]. Such combinations can relieve pain associated with acute apical periodontitis and prevent acute exacerbation of chronic apical periodontitis [32]. In the present research, one of those combinations (Ledermix) was used to study its antimicrobial effect against the three tested microorganisms. Ledermix paste contains an antibiotic, 3.2% demeclocycline–calcium, and a corticosteroid, 1% triamcinolone acetonide, in a polyethylene glycol base [33]. Both triamcinolone and demeclocycline can diffuse through dentinal tubules and cementum to reach the periapical tissues [15]. Various studies have confirmed the effectiveness of Ledermix as an intracanal medicament [24, 34, 35].

It is currently important to look for an intracanal drug with good antimicrobial activity against variable species of endodontic microorganisms. NaOCl is known to be the best irrigating solution that has had the most active antimicrobial activity against the most possible root canal microorganisms [36]. This solution, however, has high toxicity to the tissue when it is apically extruded. The use of NaOCl gel can reduce the risk of apical extrusion and the overall side effects of its solution form [37, 38]. Up to our knowledge, there is no dental company producing the gel form of NaOCl. However, the detergent companies produce this type of NaOCl as a bleaching material for clothes. Therefore, this study was conducted to evaluate and compare the antibacterial effect of sodium hypochlorite gel (Clorox Bleach Pen Gel) as a proposed intracanal medicament with that of the currently available root canal medicament such as chlorhexidine gel, calcium hydroxide paste, calcium hydroxide–iodoform combination (Diapex Plus), and antibiotic–corticosteroid paste (Ledermix).

In the present research, the agar diffusion test was chosen to assess the antimicrobial activity of the experimental medicaments because it is a simple technique by which the antimicrobial activity of endodontic materials can initially be assessed before carrying out more advanced tests [19]. It allows direct comparisons of the antimicrobial activity of endodontic materials by measuring the size of formed inhibition zones, indicating which material can eliminate possible microorganisms inside the root canal system. However, the size of the inhibition zones does not indicate the entire antimicrobial efficiency of the material [39]. The results may be influenced by many factors such as the chemical and physical properties of the tested material and culture medium [40]. In the current study, all attempts were made to decrease most of the variables such as type and thickness of agar medium, inoculums density, and incubation temperature. A Mueller-Hinton agar medium with a standardized thickness of 8 mm (Merck, Darmstadt, Germany) was used to ensure that all the agar plates had an equal thickness of agar [41]. The inoculum preparation was done similar to the method of Sipert et al. [42] and Asgary and Kamrani [43]. In order to ensure even distribution of the inoculums, the respective bacterial dilutions were swabbed uniformly onto the agar plates using lawn technique [44]. A well of 5 mm diameter and 4 mm depth was punched in each section using sterile glass Pasteur pipettes to ensure that the volume of each medicament was standardized [41, 44]. Finally, each zone of inhibition was measured with a millimeter ruler with an accuracy of 0.5 mm [43]. The results obtained from this in vitro test must be concluded with caution, because this test may not show the full clinical efficacy of the material being tested [45].

The null hypothesis of this study was rejected because the findings showed that there were significant differences between the tested medicaments regarding their antibacterial efficacy against all microbial strains.

The existing study revealed that NaOCl gel followed by 2% CHX gel had the uppermost antimicrobial activity against all tested microorganisms. This finding may be justified by the fact that NaOCl and CHX have a broad antibacterial spectrum and they are effective against both Gram-positive and Gram-negative bacteria [46]. However, the antibacterial effect of Ledermix against *S. aureus* was statistically similar to CHX gel and higher than Ca (OH)_2_ paste. Meanwhile, Ca (OH)_2_ has shown better efficacy against *E. faecalis* than Ledermix. Regarding Diapex Plus, it did not show any antibacterial activity against all tested microbial strains. Among the tested microorganisms, *E. faecalis* was the most sensitive microorganisms to NaOCl gel and calcium hydroxide paste, while *S. aureus* was the most sensitive microorganism to CHX gel and Ledermix.

The current results complement the results of Jurczyk et al., who found that minimal inhibitory concentrations of NaOCl gel were equal to CHX solution against Gram-negative but higher against Gram-positive bacteria [47]. Shamsi et al. concluded that 5.25% NaOCl gel exhibited the same effect as 5.25% NaOCl solution against *E. faecalis*, and it might be recommended as an efficient intracanal irrigation agent [48]. However, Oncag et al. found that the 2% CHX was significantly more powerful than 5.25% NaOCl against *E. faecalis* [49].

On the contrary, the findings of the present study have shown that NaOCl gel was significantly more effective against all bacterial strains than CHX. Vianna et al. reported that 0.5% and 1% NaOCl killed *E. faecalis* and *S. aureus*, within 20–30 minutes [50]. Other studies have shown that CHX gel was more effective against *E. faecalis* than the Ca (OH)_2_ paste [2, 51, 52] and can be used during the treatment of endodontic failure [53]. Gomes at al. showed that both gel and liquid formulation of 2% CHX eliminated *S. aureus* in 15 sec, while *E. faecalis* was killed by gel formulation in 1 min[54]. Despite the strong antibacterial action of NaOCL, one of its major drawbacks in the root canal system is high surface tension, which limits its penetration into dentinal tubules and irregularities of the root canal system [55]. Moreover, its cytotoxicity is considered a clinical problem especially when it is extruded in the periapical area.

The bactericidal activity of sodium hypochlorite is due to the release of hypochlorous acid (HOCL) when added to water. The hypochlorous acid contains active chlorine, a potent oxidizing agent that allows irreversible oxidation of −SH groups of essential enzymes and thus disrupts the metabolic functions of the bacterial cell [56]. The antibacterial effect of chlorhexidine may be explained by the interaction of the positively charged chlorohexidine molecules and negatively charged phosphate groups on the bacterial cell wall. This interaction can alter the osmotic balance of the bacterial cell and allow the chlorohexidine molecule to penetrate the bacteria and produces direct toxic effect [16]. When chlorhexidine is used in high concentrations, there is a precipitation of cytoplasm, with consequent death of the microorganism [57]. It is better, in particular situations such as retreatment cases, to use antimicrobial agents that exhibit substantivity, that is, agents that can have a therapeutic effect for a prolonged period of time [26]. It has been demonstrated that CHX preparations, due to their cationic nature, exhibit the best substantivity among all endodontic agents [26, 58]. Depending on its concentration, CHX demonstrated substantivity from 48 h to 12 weeks in different studies [59, 60]. Furthermore, it has been demonstrated that CHX, possibly due to its substantivity, may delay coronal leakage in endodontically treated teeth [30].

Some studies have revealed that *E. faecalis* was resistant to Ca (OH)_2_ medication [61]. Other studies indicated that calcium hydroxide was not very effective against endodontic biofilms [62] and bacteria penetrating dentinal tubules [63]. However, the findings of this study suggested that the calcium hydroxide paste demonstrated antimicrobial activity against all microbial strains but with a lower efficacy compared to NaOCl and CHX and this is in concurrence with the results of Ravishanker and Subba Rae [64]. Estrela et al. showed that Ca (OH)_2_ requires 60 days to produce antimicrobial activity against *E. faecalis* [65]. Zehnder et al. reported that Ca (OH)_2_ eliminated *E. faecalis* from radicular dentin after seven days [66]. Gomes et al. stated that Ca (OH)_2_ has no uniform antimicrobial activity against all microorganisms present in the root canal and its interaction with antimicrobial substances becomes necessary [67].

The antimicrobial properties of Ca (OH)_2_ have been attributed to its high pH (11–12.5) and its dissociated hydroxyl ions, which destroy bacterial cells by protein denaturation and its detrimental effect on the cytoplasmic membrane and DNA [68]. Moreover, it serves as a physical barrier that can limit the ingress of microorganisms and impede the supply of nutrients to the remaining microorganisms, thereby preventing reinfection of the root canal and delaying recontamination [69].

The lower antimicrobial activity of Ca (OH)_2_ medication compared with NaOCl and CHX might be clarified by the proton pump property of the microorganisms [61, 70] and its delayed action [65]. In addition, the buffering agents in the culture medium can decrease the antibacterial effect of calcium hydroxide [71]. Additionally, some authors found that calcium hydroxide paste was not able to kill *E. faecalis* in the presence of dentine, hydroxyapatite, and bovine serum [72].

In certain situations, the alkaline environment in the radicular dentin created by calcium hydroxide may inhibit the resorptive activity of dentinoclasts, which needs an acid environment to achieve mineral dissolution. Hence, calcium hydroxide cannot be considered a universal intracanal medicament for all cases of infected root canal systems with apical periodontitis [73]. The current findings showed that Ledermix has an antimicrobial effect against *E. coli* and *S. aureus* but with little effect against *E. faecalis*, and that matches the results of Seow [74]. However, Athanassiadis et al. showed that the Ledermix and Ca (OH)_2_ were effective against *E. faecalis* [75].

Although iodoform is incorporated to improve the antibacterial properties of Diapex Plus [76], the results of the present research showed that this medicament has no antibacterial effect. This finding corresponds to the results of other authors [77]. Some authors found that the oil paste containing calcium hydroxide was mainly lacking both ion release and antibacterial properties [78]. Asmaa et al. [79] and Ravishanker and Subba Rae [64] concluded that Vitapex paste with 30% Ca (OH)_2_ displayed the least inhibition of *E. faecalis*, while 1% CHX gel demonstrated better antibacterial efficacy.

Changes in the environment of microorganisms within the root canal system due to endodontic treatment may stimulate calcification of *E. faecalis* biofilms, as shown in a study by George et al. [80]. Those authors also reported that biofilms, formed under nutrient-rich conditions, had increased levels of calcium ions within the biofilm, while nutrient-poor conditions did not show this effect. This suggests a potential role of the mineralized matrix as a shelter for viable bacteria, which protects them from antimicrobial agents [80]. Further studies are therefore required to evaluate the antimicrobial effect of intracanal medicaments on the biofilms of different microorganisms.

A medicament that is effective in vitro against a single microbe may not necessarily be effective in vivo against the same microbe because the infected root canal consists of multiple microorganisms. Further clinical trials are required to validate the results of in vitro studies, addressing the effectiveness of medications against endodontic micro-organisms. Because of the excellent effectiveness of NaOCl gel against all tested microbial strains in the current study, it must be considered in the future clinical and laboratory researches aiming to release a strong intracanal medicament. Besides, dental manufacturers should take the present results of NaOCl gel in their considerations and improve its formula for use as an effective intracanal medicament. Until now, due to its excellent tissue biocompatibility and its intense antibacterial activity, CHX gel is considered the primary intracanal medicament that can be used effectively and safely until more laboratory and clinical studies on the NaOCl gel are conducted.

## 5. Conclusions

Within the limitations of the current in vitro study, the following conclusions can be drawn:

1. Sodium hypochlorite gel and chlorhexidine gel demonstrated the strongest antibacterial activities among tested medicaments
2. Calcium hydroxide paste exhibited antibacterial effect against all microbial strains particularly *E. faecalis*
3. Ledermix showed a better antibacterial effect particularly against *S. aureus* and *E. coli* than calcium hydroxide but with lower antibacterial activity on *E. faecalis*
4. Diapex Plus exhibited no antibacterial effect
5. Sodium hypochlorite gel may be considered as an excellent intracanal medicament, and more laboratory and clinical trials are required to validate its antimicrobial effectiveness and safety

## Figures and Tables

**Table 1:** Experimental intracanal medicaments.

Medicaments	Composition	Manufacturer	Lot. no.
Clorox Beach Pen	Sodium hypochlorite (0.5–2%), boehmite (3–7%), sodium silicate (0.5–1.5%), and sodium petroleum sulfonate (0.5–1.5%)	Clorox, Bleach Pen Gel for White, Clorox, 1221 Broadway Oakland, CA 94612, USA	137533.002 J
Consepsis scrub	2% chlorhexidine gluconate	Ultradent Products, Inc., 505 W. 10200 S. South Jordan, UT 84095, USA	B94CR
Ledermix	Demeclocycline calcium (30.21 mg/g), triamcinolone acetonide (10 mg/g), macrogol 400, macrogol 3000, zinc oxide, silicon dioxide, calcium chloride-dihydrate, trolamine, sodium calcium edetate, and sodium sulphate	Riemser Pharma GMH, An der Wiek 7, 17493 Greifswald-Insel Riems, Germany	350860
Metapaste	Calcium hydroxide, barium sulphate, and polypropylene glycol	Meta Biomed Co., Ltd,, South Korea	MPS1708281
Diapex Plus	Iodoform (35–40%), Ca (OH)2 (20–30%), and hydrophobic polydimethylsiloxane (20–30%)	DiaDent Group International, 16 Osongsaengmyeong 4-ro, Osong-eup, Heungdeok-gu, Cheongju-si, Chungcheongbuk-do, Korea	WX346100

**Table 2:** Bacterial strains used as indicators of antimicrobial activity, their source and morphotype.

Microorganisms	Source	Morphotype	Manufacturer
*Staphylococcus aureus* (*S. aureus*)	ATCC 6538	Gram-positive, cocci, facultative anaerobes	Microbiologics, Saint Cloud, Minnesota, USA
*Enterococcus faecalis* (*E. faecalis*)	ATCC 29212	Gram-positive, rod-shaped, facultative anaerobes	
*Escherichia coli* (*E. coli*)	ATCC 25922	Gram-negative, rod-shaped, facultative anaerobes	

**Table 3:** Comparisons of mean diameters of inhibition zones (mm) formed by the tested medicaments against each bacterial strain.

Medicaments	Mean diameter of growth inhibition zones (±SD)				ANOVA (*P* value)
	Plates number	*S. aureus*	*E. faecalis*	*E. coli*	
Sodium hypochlorite gel	10	30.68 ± 1.81^A1^*∗*^^	34.87 ± 1.31^A2^	31.43 ± 2.14^A1^	0.000
Chlorhexidine gel	10	27.83 ± 1.02^B1^	20.93 ± 0.77^B2^	24.40 ± 0.82^B3^	0.000
Ledermix	10	28.25 ± 1.87^B1^	9.05 ± 0.75^C2^	14.23 ± 0.53^C3^	0.000
Calcium hydroxide	10	14.45 ± 1.01^C1^	18.15 ± 0.51^D2^	12.27 ± 1.13^D3^	0.000
Diapex Plus	10	0.00 ^D^	0.00 ^E^	0.00 ^E^	—
ANOVA (*P* value)		0.000	0.000	0.000	—

^*∗*^Games–Howell post hoc test: different uppercase letters (columns) and different uppercase numbers (rows) indicate statistically significant differences (*P* < 0.05) among the tested medicaments against the same microorganism and among antimicrobial effects of each medicament against tested microorganisms, respectively.

## Data Availability

The data (measurements of inhibition zones) used to support the findings of this study will be available from the corresponding author elsayednada@yahoo.com for the researchers who meet the criteria for accessing this data. The data can be requested after the publication of this article. However, requests for the data (6/12 months) after the publication of this article will be considered by the corresponding author.
